# (*E*)-1-Bromo-4-(2-nitro­prop-1-en­yl)benzene

**DOI:** 10.1107/S1600536809048910

**Published:** 2009-11-21

**Authors:** Bailin Li

**Affiliations:** a Department of Pharmaceutical and Chemical Engineering, Taizhou College, Linhai, Zhejiang 317000, People’s Republic of China

## Abstract

The title compound, C_9_H_8_BrNO_2_, which was synthesized by the condensation of 4-bromo­benzaldehyde with nitro­ethane, possesses a *trans* configuration. The dihedral angle between the benzene ring and the mean plane of the double bond is 7.31 (3)°. The crystal structure is stabilized by short inter­molecular Br⋯O contacts [3.168 (4) Å].

## Related literature

For general background to nitro­alkenes as inter­mediates in the preparation of numerous products including insecticides and pharmacologically active substances, see: Boelle *et al.* (1998 ▶); Vallejos *et al.* (2005 ▶). For related structures, see: Boys *et al.* (1993 ▶); Mugnoli *et al.* (1991 ▶).
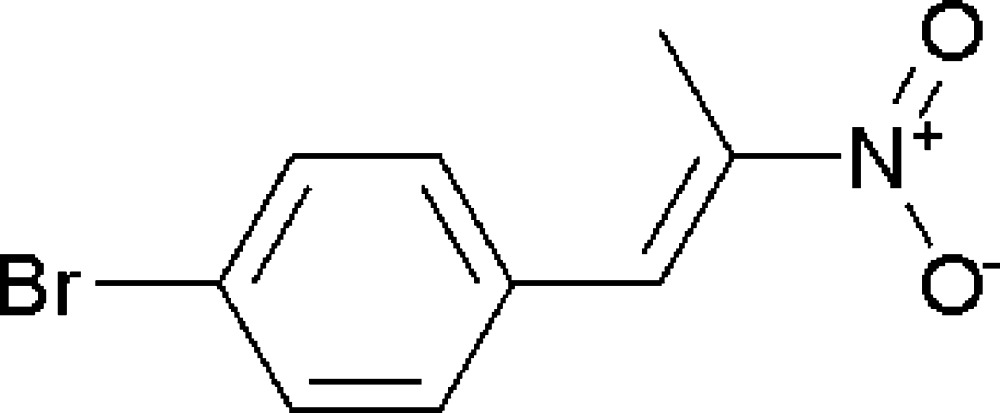


## Experimental

### 

#### Crystal data

C_9_H_8_BrNO_2_
*M* *_r_* = 242.07
Triclinic, 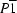

*a* = 6.9787 (5) Å
*b* = 7.4123 (5) Å
*c* = 9.7659 (6) Å
α = 105.435 (2)°
β = 95.087 (2)°
γ = 104.323 (2)°
*V* = 465.31 (5) Å^3^
*Z* = 2
Mo *K*α radiation
μ = 4.38 mm^−1^
*T* = 296 K
0.21 × 0.19 × 0.08 mm

#### Data collection

Rigaku R-AXIS RAPID diffractometer
Absorption correction: multi-scan (*ABSCOR*; Higashi, 1995 ▶) *T*_min_ = 0.388, *T*_max_ = 0.703
4605 measured reflections
2112 independent reflections
1303 reflections with *I* > 2σ(*I*)
*R*_int_ = 0.027

#### Refinement

*R*[*F* ^2^ > 2σ(*F* ^2^)] = 0.035
*wR*(*F* ^2^) = 0.094
*S* = 1.00
2112 reflections
120 parameters
H-atom parameters constrained
Δρ_max_ = 0.46 e Å^−3^
Δρ_min_ = −0.71 e Å^−3^

### 

Data collection: *PROCESS-AUTO* (Rigaku, 2006 ▶); cell refinement: *PROCESS-AUTO*; data reduction: *CrystalStructure* (Rigaku/MSC, 2007 ▶); program(s) used to solve structure: *SHELXS97* (Sheldrick, 2008 ▶); program(s) used to refine structure: *SHELXL97* (Sheldrick, 2008 ▶); molecular graphics: *ORTEP-3 for Windows* (Farrugia, 1997 ▶); software used to prepare material for publication: *WinGX* (Farrugia, 1999 ▶).

## Supplementary Material

Crystal structure: contains datablocks global, I. DOI: 10.1107/S1600536809048910/zq2017sup1.cif

Structure factors: contains datablocks I. DOI: 10.1107/S1600536809048910/zq2017Isup2.hkl

Additional supplementary materials:  crystallographic information; 3D view; checkCIF report

## Figures and Tables

**Fig. 1.**
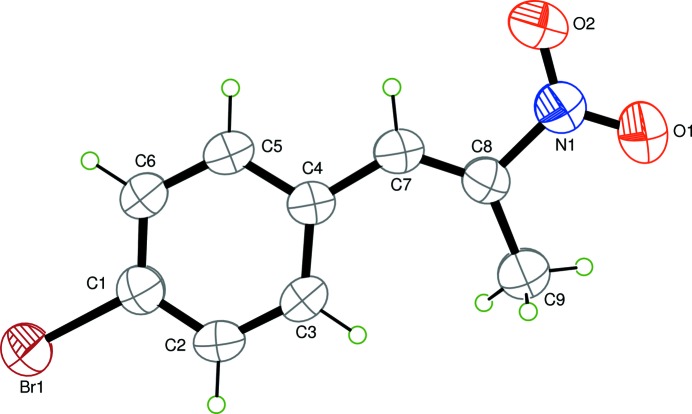
The asymmetric unit of the title compound (I) with the atomic labeling scheme. Displacement ellipsoids are drawn at the 40% probability level.

**Fig. 2.**
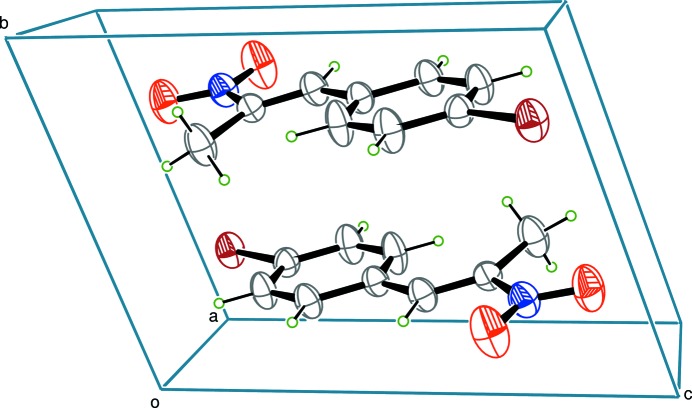
Molecular packing of the title compound (I) viewed down the a axis.

## supplementary crystallographic information

### Comment

Nitroalkenes are valuable intermediates for preparation of numerous products including insecticides and pharmacologically active substances (Boelle *et al.*, 1998 and Vallejos *et al.*, 2005) in which the nitro group can be easily transformed into a variety of groups with different functionalities, such as amine, carbonyl groups, *etc*.. In this article, the crystal structure of the title compound (*E*)-1-bromo-4-(2-nitroprop-1-enyl)benzene is presented (Fig. 1). The dihedral angle between the benzene ring and the mean plan of the double bond H7/C7/C8/C9 is 7.31 (3) °. The crystal structure is stabilized by short intermolecular Br—O contacts [3.168 (4) Å].

### Experimental

To a solution of 4-bromobenzaldehyde (50 mmol) in AcOH (25 ml), nitroethane (75 mmol) was added, followed by butylamine (100 mmol, 7.4 ml). The mixture was sonicated at 333 K, until TLC showed full conversion of aldehyde. The mixture was poured into ice water, the precipitate was filtered off, washed with water and recrystallized from EtOH to give (*E*)-1-bromo-4-(2-nitroprop-1-enyl)benzene. Suitable crystals of the title compound were obtained by slow evaporation of an ethanol solution at room temperature.

### Refinement

All carbon-bonded H atoms were placed in calculated positions with C—H = 0.93 Å (aromatic), C—H = 0.96 Å (*sp*) and refined using a riding model, with *U*_iso_(H) = 1.2_eq_(C).

### Crystal data

**Table:**

C_9_H_8_BrNO_2_	*Z* = 2
*M**_r_* = 242.07	*F*(000) = 240
Triclinic, *P*1	*D*_x_ = 1.728 Mg m^−^^3^
Hall symbol: -P 1	Mo *K*α radiation, λ = 0.71073 Å
*a* = 6.9787 (5) Å	Cell parameters from 3184 reflections
*b* = 7.4123 (5) Å	θ = 3.1–27.4°
*c* = 9.7659 (6) Å	µ = 4.38 mm^−^^1^
α = 105.435 (2)°	*T* = 296 K
β = 95.087 (2)°	Platelet, yellow
γ = 104.323 (2)°	0.21 × 0.19 × 0.08 mm
*V* = 465.31 (5) Å^3^	

### Data collection

**Table:**

Rigaku R-AXIS RAPID diffractometer	2112 independent reflections
Radiation source: rolling anode	1303 reflections with *I* > 2σ(*I*)
graphite	*R*_int_ = 0.027
Detector resolution: 10.00 pixels mm^-1^	θ_max_ = 27.4°, θ_min_ = 3.1°
ω scans	*h* = −9→9
Absorption correction: multi-scan (*ABSCOR*; Higashi, 1995)	*k* = −9→8
*T*_min_ = 0.388, *T*_max_ = 0.703	*l* = −12→12
4605 measured reflections	

### Refinement

**Table:**

Refinement on *F*^2^	Secondary atom site location: difference Fourier map
Least-squares matrix: full	Hydrogen site location: inferred from neighbouring sites
*R*[*F*^2^ > 2σ(*F*^2^)] = 0.035	H-atom parameters constrained
*wR*(*F*^2^) = 0.094	*w* = 1/[σ^2^(*F*_o_^2^) + (0.012*P*)^2^ + 0.950*P*] where *P* = (*F*_o_^2^ + 2*F*_c_^2^)/3
*S* = 1.00	(Δ/σ)_max_ < 0.001
2112 reflections	Δρ_max_ = 0.46 e Å^−^^3^
120 parameters	Δρ_min_ = −0.71 e Å^−^^3^
0 restraints	Extinction correction: *SHELXL97* (Sheldrick, 2008), Fc^*^=kFc[1+0.001xFc^2^λ^3^/sin(2θ)]^-1/4^
Primary atom site location: structure-invariant direct methods	Extinction coefficient: 0.0149 (13)

### Special details

**Table:**

Geometry. All e.s.d.'s (except the e.s.d. in the dihedral angle between two l.s. planes) are estimated using the full covariance matrix. The cell e.s.d.'s are taken into account individually in the estimation of e.s.d.'s in distances, angles and torsion angles; correlations between e.s.d.'s in cell parameters are only used when they are defined by crystal symmetry. An approximate (isotropic) treatment of cell e.s.d.'s is used for estimating e.s.d.'s involving l.s. planes.
Refinement. Refinement of *F*^2^ against ALL reflections. The weighted *R*-factor *wR* and goodness of fit *S* are based on *F*^2^, conventional *R*-factors *R* are based on *F*, with *F* set to zero for negative *F*^2^. The threshold expression of *F*^2^ > σ(*F*^2^) is used only for calculating *R*-factors(gt) *etc*. and is not relevant to the choice of reflections for refinement. *R*-factors based on *F*^2^ are statistically about twice as large as those based on *F*, and *R*- factors based on ALL data will be even larger.

### Fractional atomic coordinates and isotropic or equivalent isotropic displacement parameters (Å^2^)

**Table:**

	*x*	*y*	*z*	*U*_iso_*/*U*_eq_	
Br1	0.90197 (7)	0.24927 (9)	0.09032 (5)	0.0754 (2)	
N1	0.2076 (6)	0.2311 (6)	0.7592 (4)	0.0660 (10)	
O1	0.2341 (5)	0.2609 (6)	0.8891 (4)	0.0937 (12)	
O2	0.0411 (5)	0.1719 (7)	0.6875 (4)	0.1038 (14)	
C8	0.3870 (6)	0.2659 (6)	0.6875 (4)	0.0514 (9)	
C1	0.7334 (6)	0.2408 (6)	0.2327 (4)	0.0567 (10)	
C5	0.4097 (6)	0.1824 (7)	0.2989 (4)	0.0631 (12)	
H5	0.2712	0.1456	0.2720	0.076*	
C7	0.3513 (6)	0.2154 (7)	0.5461 (4)	0.0594 (11)	
H7	0.2165	0.1633	0.5051	0.071*	
C4	0.4907 (6)	0.2283 (6)	0.4430 (4)	0.0524 (10)	
C3	0.6976 (6)	0.2770 (8)	0.4767 (5)	0.0776 (15)	
H3	0.7569	0.3055	0.5720	0.093*	
C9	0.5777 (7)	0.3501 (9)	0.7900 (5)	0.0825 (16)	
H9A	0.6349	0.2476	0.7987	0.099*	
H9B	0.5528	0.4165	0.8825	0.099*	
H9C	0.6694	0.4409	0.7555	0.099*	
C2	0.8179 (6)	0.2840 (8)	0.3723 (5)	0.0742 (14)	
H2	0.9565	0.3183	0.3976	0.089*	
C6	0.5299 (6)	0.1901 (7)	0.1943 (4)	0.0680 (13)	
H6	0.4727	0.1609	0.0985	0.082*	

### Atomic displacement parameters (Å^2^)

**Table:**

	*U*^11^	*U*^22^	*U*^33^	*U*^12^	*U*^13^	*U*^23^
Br1	0.0560 (3)	0.1058 (5)	0.0598 (3)	0.0136 (2)	0.0153 (2)	0.0237 (3)
N1	0.055 (2)	0.081 (3)	0.055 (2)	0.0147 (19)	0.0103 (18)	0.014 (2)
O1	0.076 (2)	0.142 (4)	0.056 (2)	0.019 (2)	0.0205 (17)	0.025 (2)
O2	0.0467 (19)	0.173 (4)	0.074 (2)	0.017 (2)	0.0114 (17)	0.020 (2)
C8	0.045 (2)	0.056 (3)	0.050 (2)	0.0112 (18)	0.0092 (17)	0.0142 (19)
C1	0.052 (2)	0.066 (3)	0.052 (2)	0.013 (2)	0.0100 (19)	0.019 (2)
C5	0.042 (2)	0.087 (3)	0.050 (2)	0.012 (2)	−0.0015 (18)	0.012 (2)
C7	0.043 (2)	0.077 (3)	0.051 (2)	0.015 (2)	0.0032 (17)	0.012 (2)
C4	0.042 (2)	0.064 (3)	0.048 (2)	0.0140 (19)	0.0039 (16)	0.013 (2)
C3	0.050 (2)	0.134 (5)	0.044 (2)	0.024 (3)	−0.0013 (19)	0.022 (3)
C9	0.052 (3)	0.122 (5)	0.055 (3)	0.008 (3)	0.003 (2)	0.015 (3)
C2	0.038 (2)	0.123 (4)	0.055 (3)	0.018 (2)	0.0015 (19)	0.022 (3)
C6	0.047 (2)	0.103 (4)	0.045 (2)	0.013 (2)	0.0013 (18)	0.017 (2)

### Geometric parameters (Å, °)

**Table:**

Br1—C1	1.902 (4)	C7—C4	1.466 (5)
N1—O2	1.214 (5)	C7—H7	0.9300
N1—O1	1.217 (4)	C4—C3	1.385 (6)
N1—C8	1.488 (5)	C3—C2	1.380 (6)
C8—C7	1.314 (5)	C3—H3	0.9300
C8—C9	1.478 (6)	C9—H9A	0.9600
C1—C2	1.357 (6)	C9—H9B	0.9600
C1—C6	1.366 (6)	C9—H9C	0.9600
C5—C6	1.381 (6)	C2—H2	0.9300
C5—C4	1.388 (5)	C6—H6	0.9300
C5—H5	0.9300		
			
O2—N1—O1	122.1 (4)	C5—C4—C7	117.7 (4)
O2—N1—C8	119.7 (4)	C2—C3—C4	121.6 (4)
O1—N1—C8	118.2 (4)	C2—C3—H3	119.2
C7—C8—C9	130.9 (4)	C4—C3—H3	119.2
C7—C8—N1	115.8 (4)	C8—C9—H9A	109.5
C9—C8—N1	113.2 (3)	C8—C9—H9B	109.5
C2—C1—C6	120.5 (4)	H9A—C9—H9B	109.5
C2—C1—Br1	119.2 (3)	C8—C9—H9C	109.5
C6—C1—Br1	120.3 (3)	H9A—C9—H9C	109.5
C6—C5—C4	121.6 (4)	H9B—C9—H9C	109.5
C6—C5—H5	119.2	C1—C2—C3	119.9 (4)
C4—C5—H5	119.2	C1—C2—H2	120.0
C8—C7—C4	130.1 (4)	C3—C2—H2	120.0
C8—C7—H7	115.0	C1—C6—C5	119.5 (4)
C4—C7—H7	115.0	C1—C6—H6	120.2
C3—C4—C5	116.9 (4)	C5—C6—H6	120.2
C3—C4—C7	125.4 (4)		
			
O2—N1—C8—C7	−4.8 (6)	C8—C7—C4—C5	−173.5 (5)
O1—N1—C8—C7	174.2 (5)	C5—C4—C3—C2	1.5 (8)
O2—N1—C8—C9	176.1 (5)	C7—C4—C3—C2	179.4 (5)
O1—N1—C8—C9	−4.9 (6)	C6—C1—C2—C3	−0.1 (8)
C9—C8—C7—C4	−0.7 (9)	Br1—C1—C2—C3	−179.5 (4)
N1—C8—C7—C4	−179.7 (4)	C4—C3—C2—C1	−0.6 (8)
C6—C5—C4—C3	−1.8 (7)	C2—C1—C6—C5	−0.2 (8)
C6—C5—C4—C7	−179.8 (4)	Br1—C1—C6—C5	179.2 (4)
C8—C7—C4—C3	8.7 (8)	C4—C5—C6—C1	1.1 (8)
